# A Novel Deep Learning Approach for Recognizing Stereotypical Motor Movements within and across Subjects on the Autism Spectrum Disorder

**DOI:** 10.1155/2018/7186762

**Published:** 2018-07-10

**Authors:** Lamyaa Sadouk, Taoufiq Gadi, El Hassan Essoufi

**Affiliations:** Faculty of Science and Technology, University Hassan 1, Settat, Morocco

## Abstract

Autism Spectrum Disorder (ASD) is a neurodevelopmental disorder characterized by persistent difficulties including repetitive patterns of behavior known as stereotypical motor movements (SMM). So far, several techniques have been implemented to track and identify SMMs. In this context, we propose a deep learning approach for SMM recognition, namely, convolutional neural networks (CNN) in time and frequency-domains. To solve the intrasubject SMM variability, we propose a robust CNN model for SMM detection within subjects, whose parameters are set according to a proper analysis of SMM signals, thereby outperforming state-of-the-art SMM classification works. And, to solve the intersubject variability, we propose a global, fast, and light-weight framework for SMM detection across subjects which combines a knowledge transfer technique with an SVM classifier, therefore resolving the “real-life” medical issue associated with the lack of supervised SMMs per testing subject in particular. We further show that applying transfer learning across domains instead of transfer learning within the same domain also generalizes to the SMM target domain, thus alleviating the problem of the lack of supervised SMMs in general.

## 1. Introduction

Autism Spectrum Disorder ASD refers to a spectrum of disorders with a range of manifestations that can occur on different degrees and in a variety of forms [1]. Children with ASD have impairments in social interaction and communication as well as atypical behaviors that include repetitive behaviors known as stereotypical motor movements (SMM). The most prevalent SMMs include repetitive body rocking, mouthing, and complex hand and finger movements [2] which interfere tremendously with learning and social interactions, thus reducing school and community integration [3, 4]. Inasmuch as they are largely resistant to psychotropic drugs, decreasing or eliminating SMMs is the goal of many behavioral interventions in autism. And the earlier the age at which intervention can be started is, the better their learning and daily function can be facilitated [5, 6].

To address this challenge, the role of sensing technologies becomes critical in identifying SMMs throughout the screening and therapy of ASD, thus potentially improving the lives of those in the spectrum. Indeed, reliably and efficiently detecting and monitoring SMMs over time could be beneficial not only for recognizing autistic children but also for understanding and intervening upon a core symptom of ASD. Efficient and accurate monitoring of SMM is needed to (i) continuously evaluate which therapies and/or drugs are required over time and (ii) identify mechanisms that are responsible for triggering an SMM, such as physiological, affective, or environmental factors. Such monitoring can help therapists reduce the frequency of SMMs and gradually lower their duration and severity [4].

The main problematic for a perfect SMM recognition is to find the most relevant features that characterize stereotypical behaviors from accelerometer signals through an automatic feature extraction technique. Another problematic is personalization caused by the intra- and intersubject variability [7, 8]. Indeed, intrasubject variances can be explained by the variations in intensity, duration, and frequency of SMMs within each atypical subject (subject on the autism spectrum) while intersubject variances are due to differences in SMMs across different atypical subjects. Hence, an adaptive approach is needed to generalize over all SMMs within and across subjects and adjust to new SMM behaviors. These problematics can be addressed using deep learning techniques for SMM recognition. Our contributions are the following: (i) for SMM detection within subjects, we train two CNN models in time and frequency-domains whose parameters are chosen based on properties of the input SMM signals, (ii) for SMM detection across subjects, we build a global, fast and light-weight platform combining a knowledge transfer platform to an SVM classifier, which provides promising results by adapting to SMMs of any new atypical subject. One advantage of this platform is the use of only few labeled data of this atypical subject as well as a large data of some other atypical subjects, thus resolving the “real-life” medical issue associated with the lack of supervised SMMs per atypical subject, (iii) applying cross-domain transfer learning (i.e., coming from a source domain different from the SMM target domain) instead of within-domain transfer learning provides a satisfying SMM recognition performance especially in time-domain and thus adjusts to stereotypical behavior patterns of any new atypical subject with only few of his labeled data.

In the section below, we review related works of SMM recognition (Section 2). Then, we describe our methodology, namely, our proposed deep learning model for SMM recognition within subjects as well as our scalable platform for SMM recognition across subjects (Section 3). In Section 4, a description of the datasets used is given, the data preprocessing is detailed, and the CNN architecture structure is defined. In Section 5, experiments are explained and carried out, and corresponding results are laid out. Finally, Section 6 concludes our work.

## 2. Related Work

Several studies have been conducted in order to detect SMM behaviors in individuals with ASD. Traditional methods of SMM relied primarily on paper-and-pencil rating scales, direct behavioral observation, and video-based coding, all of which have limitations. While paper-and-pencil rating scales and direct behavioral observation may provide some unreliable and invalid measures, video-based methods provide more accurate and reliable measures but are time consuming because experts have to view videos repeatedly with slow playback speeds.

Automatic approaches that are time efficient and more accurate have been proposed. These approaches rely on wireless accelerometers and pattern recognition algorithms. Existing approaches to automated monitoring of SMM are based on either webcams or accelerometers. In a series of publications [9–11], Gonçalves et al. make use of the Kinect webcam sensor (from Microsoft) to detect only one type of SMM, namely, hand flapping. However, the Kinect sensor is limited to monitoring within a confined space and requires users to be in close proximity to the sensor.

Alternative approaches to the Kinect are based on the use of one or more wearable 3-axis accelerometers (located in wrist or torso for instance). Using three, three-axis accelerometers sampling at 100Hz, Westeyn et al. [12] recognized 69% of hand flapping events using Hidden Markov Models; however, data were acquired from healthy individuals mimicking behaviors. A similar work that did not generalize to the ASD population was the study of Plötz et al., 2012 [13]. Meanwhile, Min et al. [14–17] reports collected a total of 40 hours of three-axis acceleration data from the wrists and torso of four individuals with autism. Using a variety of different features and semisupervised classification approaches (Orthogonal Matching Pursuit, Liner Predictive Coding, all-pole Autoregressive model, Higher Order Statistics, Ordinary Least Squares, K-VSD algorithm), they achieved uppermost accuracy rates of 86% for hand flapping and 95% for body rocking; however, true positive and false positive rates were not adequately described. Goodwin et al. [7, 18–20] collected three-axis acceleration data from the wrists and torso of six individuals with autism repeatedly observed in both laboratory and classroom settings. By combining time and frequency-domain features (distances between mean values along accelerometer axes, variance along axes directions, correlation coefficients, entropy, Fast Fourier Transform (FFT) peaks, and Stockwell transform) to C4.5 decision, SVM, and DT classifiers, they were able to achieve an overall accuracy of 81.3% on SMM detection. And recently, Großekathöfer et al. [8] applied Goodwin's benchmark to introduce a new set of features based on recurrence plot and quantification analysis along with Decision Trees and Random Forest classifiers to obtain an accuracy slightly better than [7].

Nonetheless, features of all these previous publications have several limitations: (i) they are mainly aimed at characterizing oscillatory features of SMMs as statistical characteristics, joint relation of changes in different axial directions, or frequency components of oscillatory moves. Therefore, these features cannot capture dynamics of SMM that can change over time, namely, when they do not follow a consistent oscillatory pattern or when patterns differ in frequency, duration, speed, and amplitude, (ii) each and every study has a sensor type different from the other with different sensor orientations, resulting in features with different values even though they characterize the same SMM behavior, (iii) these hand-crafted features seriously depend on parameters of random combinations of features and models selected by experts and researchers, which is computationally and timely expensive.

To overcome these limitations, Rad et al. [21] proposed features learned by deep convolutional neural networks, which performed higher than traditional learning methods in SMM detection within subjects (i.e., within each subject independently). However, because of intersubject variability (i.e., pattern variations across subjects), models of this deep learning work and all previous works are built for each and every subject based on a large dataset of labeled observations per subject. So, up to now, there is no general system that adapts to SMMs of all subjects. Accordingly, we propose a deep learning system that not only learns better discriminating features for SMM pattern recognition but also is robust to intersubject variability and adapts rapidly to SMMs across subjects with only few labeled observations per subject. We hypothesize that feature learning using deep learning models such as CNNs and transfer knowledge capabilities of these models provide more accurate SMM detectors, as well as a platform for tracking not only SMMs of different record sessions within the same atypical subject but also SMMs of different atypical subjects.

## 3. Methodology

In this section, we introduce the key components for training our deep learning models. First, we first define the SMM detection problem and describe how SMM data is extracted and converted into input signals (Section 3.1). Next, we present a background on our deep learning model, namely, the CNN (Section 3.2).  Section 3.3 explains how the optimal parameters of CNN models are chosen based on an analysis of the input signals. Finally, Section 3.4 defines the global light-weight platform that adapts to SMMs of any atypical subject.

### 3.1. The SMM Detection Problem and Data Extraction

The SMM dataset consists of time-series data that are composed of multiple channels D, i.e., x, y, and z coordinate measurements recorded from multiple sensors/devices. The first step is to convert these D-channel raw data into multiple fixed-length signals in both time and frequency-domain, which will be denoted as frames. The second step is to normalize these latter before being fed into our deep learning system.

#### 3.1.1. Extraction of Dataset Signals

In order to extract* time-domain* frames, raw data, which represent multiple time-series of length *T* each (one time-series per channel), are segmented with a fixed-length sliding window *τ* and with a high overlap rate of *R* between consecutive data segments, which means that the sliding window moves with *S* time steps where *S* = *floor*(*τ* × (1 − *R*)). This results in *N* frames (i.e., N samples), where *N* = *floor*((*T* − *τ*)/*S*) + 1 and each frame is a time-point sample of size *τ* × 1 × *D*, *D* referring to the number of channels. Thus the extracted data **X** is a *τ* × 1 × *D* × *N* matrix.

In order to extract* frequency-domain* frames, time-series data are converted into frequency signals using Stockwell transform (ST) [22, 23] instead of the Fast Fourier Transform (FFT) which is restricted to a predefined fixed window length. Unlike FFT, ST adaptively captures spectral changes over time without windowing of data and therefore results in a better time-frequency resolution for nonstationary signals [22].

Let *h*[*l*] = *h*(*l* · *T*), *l* = 0,1, ⋯ , *N* − 1, be the samples of the continuous signal *h*(*t*), where *T* is the sampling interval (i.e., the sampling interval of our sensor or measuring device). The Discrete Fourier Transform (DFT) is given by

(1) $$\begin{array}{c} H\left[\;m\;\right]=\overset{N-1}{\underset{l=0}{\sum }}h\left[\;l\;\right]e^{-i2\pi ml/N} \end{array}$$

where the discrete frequency index *m* = 0,1, ⋯ , *N* − 1.

The Discrete Stockwell Transform (DST) is obtained using the following formula:

(2) $$\begin{array}{c} S\left[\;k,n\;\right]=\overset{N-1}{\underset{m=0}{\sum }}W\left[\;m\;\right]H\left[\;m+n\;\right]e^{i2\pi mk/N} \end{array}$$

where *k* is the index for time translation and *n* is the index for frequency shift. The function *W*[*m*] = *e*^−2*π*^2^*m*^2^/*n*^2^^ is the Gaussian window in the frequency-domain.

For a signal of length *N*, the numerical implementation of the DST can be summarized as below:

1. Apply an *N*-point DFT to calculate the Fourier spectrum of the signal H[m].
2. Multiply *H*[*m* + *n*] with the Gaussian window function *W*[*m*] = *e*^−2*π*^2^*m*^2^/*n*^2^^.
3. For each fixed frequency shift *n* = 0,1, ⋯ , *τ* − 1 (where *τ* is the number of frequency steps desired), apply an *N*-point inverse DFT to *W*[*m*]*H*[*m* + *n*] in order to calculate the DST coefficients [*k*, *n*], where *k* = 0,1, ⋯ , *N* − 1.

Note that there is a DST coefficient calculated for every pair of 〈*k*, *n*〉 in the time-frequency-domain. Therefore, the result of the DST is a complex matrix of size *τ* × *N* where the rows represent the frequencies for every frequency shift index *n* and the columns are the time values of every time translation index *k*; i.e., each column is the “local spectrum” for that point in time.

Knowing that our time-series data is composed of multiple channels *D*, we end up with *D* DST matrices, which can be represented as a single *τ* × 1 × *D* × *N* matrix **X**.

#### 3.1.2. Normalization

Since each of the data instances has *D* channels, each channel has to be normalized separately. A channel-wise normalization is performed to scale all values to [-1  1] according to the formula $x_{normalized}=\left(x-\bar{x}\right)/\sigma$, where *x* is the data in a specific channel, $\bar{x}$ the mean of that channel, and *σ* its standard deviation.

### 3.2. Research Background on Convolutional Neural Networks

Given an input **X**_*t*_ (**X**_*t*_ ∈ **X) **which is a *τ* × 1 × *D* matrix representing one acceleration signal at time *t*, our CNN model will predict the probability of being an SMM *P*  (*y* = 2∣**X**_*t*_), *y* = 2 corresponding to an SMM sample, and the probability of being a non-SMM *P*  (*y* = 1∣**X**_*t*_).

CNN is regarded as a specialized type of neural networks which updates weights at each layer of the visual hierarchy during training via a back propagation mechanism [24]. CNN benefits from invariant local receptive fields, shared weights, and spatiotemporal subsampling features to provide robustness over shifts and distortions in the input space [25]. A classic CNN has a hierarchical architecture that alternates between convolutional and pooling layers in order to summarize large input spaces with spatiotemporal relations into a lower dimensional feature.

*Convolution*. Each convolutional layer performs a 1D convolution on its input maps followed by an activation function, generally a rectified linear unit (ReLU), to add nonlinearity to the network and also to avoid the gradient vanishing problem. The output feature maps are generated for each convolution layer and the resultant value of unit at position *i* in the *j*th feature map in the *l*th layer, denoted as *v*_*i*_^*l*,*j*^, is given by

(3) $$\begin{array}{c} v_{i}^{l,j}=\sigma \left(\;b_{j}^{l}+\underset{k}{\sum }\overset{M}{\underset{m=1}{\sum }}w_{mk}^{l,j}v_{i+m-1}^{l-1,j}\;\right) \end{array}$$

where *σ* is the activation function, *b*_*j*_^*l*^ is the bias term for the *j*th feature map of the *l*th convolutional layer, *k* indexes over the set of feature maps in the (*l* − 1)th layer connected to the current feature map, *m* is kernel index, *M* is the filter size (width of the convolutional kernel), and *w*_*mk*_^*l*,*j*^ is the weight or value at the position *i* of the kernel connected to the *k*th feature map.

*Max-Pooling*. In order to reduce the sensitivity of the output to shifts and distortions, *v*_*i*_^*l*,*j*^ is passed through a pooling layer which performs a subsampling over a pooling window with size of R elements and calculates the reduced feature map. The output feature map *p*_*i*_^*l*,*j*^ is achieved by computing maximum values over nearby inputs of the feature map:

(4) $$\begin{array}{c} p_{i}^{l,j}=\underset{r\in R}{\max}\left(\;v_{i\times T+r}^{l,j}\;\right) \end{array}$$

where *R* is the pooling size and *T* is the pooling stride.

*Output*. The output of these layers generated by stacking several convolutional and pooling layers is flattened as a vector, a feature vector *p* = [*p*_1_, …, *p*_*I*_] which is then fed into a fully connected layer (F1), and an activation layer to produce the following output:

(5) $$\begin{array}{c} p_{i}^{l}=\underset{j}{\sum }w_{ji}^{l-1}\times \left(\;\sigma \left(\;p_{i}^{l-1}\;\right)+b_{i}^{l-1}\;\right) \end{array}$$

where *σ* is the activation function, *w*_*ji*_^*l*−1^ is the weight connecting the *i*^th^ node on layer *l* − 1 and the *j*^th^ node on layer *l*, and *b*_*i*_^*l*−1^ is the bias term.

**Figure 1:**
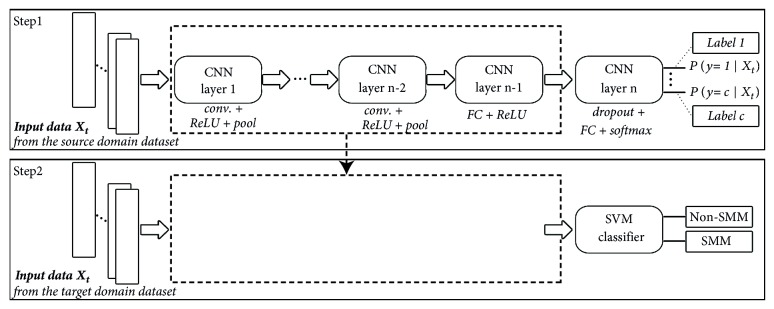
The overall transfer learning framework. “conv.” and “FC” stand for convolutional and fully connected layers, respectively.

Then a dropout layer is added to prevent the neural network from overfitting.  Figure 1 shows the fully connected neural network with dropout (F1).

Finally, this output is fed into a second fully connected layer (*F*2) and a softmax layer which infers the activity class:

(6) $$\begin{array}{c} P\left(\;c\;|\;p\;\right)=\frac{\exp\left(p_{c}^{L}\right)}{\sum_{k=1}^{N}exp\left(p_{k}^{L}\right)} \end{array}$$

where *c* is the activity class, *L* is the last layer index, and *N* is the total number of activity classes. This softmax function provides the posterior probability of the classification results. Then, an entropy cost function can be constituted based on the true labels of training instances and probabilistic outputs of softmax function.

### 3.3. Analyzing SMM Signals and Selecting the Best CNN Parameters

In convolutional neural networks (CNN), multiple hyperparameters are to be chosen carefully in order to maximize the performance of the network and retrieve the best classification rate. There are two types of hyperparameters: (i) hyperparameters of the Approximate Optimization such as the learning rate, momentum, minibatch size, and number of training iterations, (ii) and hyperparameters of the model and training criterion such as the number of hidden layers, their size (i.e., number of feature maps), the filter size of convolutional layers, and the regularizer (dropout).

In our study, we focus on hyperparameters of the CNN model, i.e., the architecture of the CNN. Generally, there is no general rule as to how to design the architecture of CNNs. Indeed, for some parameters like the number of feature maps, the trial-and-error process is implemented whereby several experiments are run by varying their values, and the optimal value will be the one that gives/yields the best classification rate. However, some other parameters such as the filter size of convolutional layers and the number of hidden layers can be determined based on the initial configuration of the input such as properties of variations present within the input signals.

#### 3.3.1. Filter Size of the 1^st^ Convolutional Layer

One of the parameters that can be determined is the filter size of the 1^st^ convolutional layer of the CNN. Indeed, choosing the proper size of the 1^st^ layer filters generates features maps which are more informative about the input signal and which capture the best fluctuations within the input signal. A small filter convolves only a part of a signal peak and therefore cannot detect a whole fluctuation within the signal, while a large filter convolves 2 to 3 signal peaks at once and therefore no fluctuation is detected either. The best filter size is the one that convolves an entire signal peak. Therefore, it is necessary to find the optimal length of all signal peaks present within input signals. To do so, we apply* sampling*, a statistical procedure that is concerned with the selection of some observations to help us make statistical inferences about the whole population. In inferential statistics, we want to use characteristics of the sample (i.e., sample peak lengths taken from randomly selected signals) to estimate the characteristics of the population (i.e., the optimal peak length of all signals). The sample statistic can be the mean, median, or mode. In our study, we choose either (i) the sample mean or (ii) the sample median (the statistic) to estimate the population mean and median, respectively.

*(i) Sample Mean.* The statistical estimation theorem states that if we have a population with mean *μ* and take one sample with *n* random values **x** (*n* being sufficiently large: *n* ≥ 30) from the population with replacement, then the distribution of these sample values will be approximately normally distributed and its mean E(**x**) is a point estimate of *μ* and E(**x**) = *μ*, where E(**x**) = (1/*n*)∑_*i*=1_^*n*^*x*_*i*_. However, using the sample mean E(**x**) as the optimal peak length and as the size of the 1^st^ convolutional layer filters does not yield good SMM recognition since it is influenced by extremes values (i.e., by signals peaks whose length are either too small and too big). In this case, we use the sample median.

*(ii) Sample Median*. For the sample median *M*_*e*_(**x**) to be a point estimate of the population median *M*_*e*_ (with a small bias), the distribution of the sample values **x** should be normally distributed. Nonetheless, the asymptotic distribution of the sample median in the classical definition is well-known to be normal for absolutely continuous distributions only and not for discrete distributions like ours. So, the statistical estimation theorem does not hold for computing the sample median of our discrete distribution. But, it has been found that the definition of the sample median based on mid-distribution functions resolves this issue and provides a unified framework for asymptotic properties of the sample median from discrete distributions [26]. Therefore, the sample median, as defined through the concept of “mid-distribution function”, has an asymptotically normal distribution, which implies that *M*_*e*_(**x**) ≈ *M*_*e*_.

#### 3.3.2. Depth of the Network

The number of hidden layers depends on several parameters including the value of dropout, the size of the convolutional layer filters, and the size of the input signal. Indeed, choosing a high dropout suggests the need for a deeper architecture. Also, the choice of the filter size of the 1^st^ convolutional layer affects the depth of the network. In other words, once the size of this filter has been set, the size (height and width) of the resultant feature maps will determine the number of extraconvolutional layers that could be stacked on top of the 1^st^ layer. For instance, a larger filter results in less hidden layers. Moreover, the bigger the size of the input signal, the more convolutional and subsampling layers can be added, resulting in more hidden layers.

### 3.4. Feature Learning via Knowledge Transfer

Many cognitive tasks require reusing knowledge acquired in one domain to perform one or more tasks in a someway related domain, without needing to learn the new task completely from scratch. In particular, given a source domain with a source learning task and a target domain with a target learning task, knowledge transfer aims to improve learning of the target predictive function using the knowledge in the source domain and source task, with the assumption that source and target domains share a common feature space [27]. In our study the target domain is the SMM dataset of an atypical subject *i* while the source domain is either similar to the target domain (such as SMM datasets of multiple atypical subjects other than *i*) or different but related to it (such as datasets of human activities performed by typical subjects). So, the purpose of this section is to prove that some of the complex features emerging from discriminative learning of our CNN models (time-domain and frequency-domain CNNs) in one of the 2 source domains can be successively used via transfer learning to classify SMM patterns of any subject *i*.

#### 3.4.1. Source Domains

To detect SMMs of an atypical subject *i*, feature learning is performed according to Step1 of Figure 1, whereby features are learned by training a CNN using data from 2 source domains. We consider

1. features learned from SMMs of some atypical subjects other than *i* as the first source domain, which are employed by a transfer learning system to recognize SMMs of new subjects. The knowledge transfer process will be conducted within the same domain where the source domain consists of SMMs of some atypical subjects and the target domain consists also of SMMs but from a different atypical subject;
2. features learned from standard movements of normal individuals (such as walking, sitting, standing, jumping, and running) taken from everyday life basis as the second source domain, which are then applied to recognize SMMs via knowledge transfer. Therefore, this latter is a process performed across two different but related domains, where the source domain is composed of simple human activities of typical subjects and the target-domain of SMMs of atypical subjects.

#### 3.4.2. Knowledge Transfer

Once features are learned via CNN from a source domain which is either SMMs in case (1) or simple human activities in case (2), they are further used via knowledge transfer to identify instances of the target domain. In particular, the CNN low- and mid-level features are used (kept unchanged) while high-level features are discarded and replaced by new randomly initialized ones. Forming/building our transfer learning framework consists of adding on top of the low- and mid-level features a “readout module” which maps the representation of these low- and mid-level features, given some input patterns, into 2 labels: SMM or non-SMM. For our readout module, a simple classifier is implemented, which is the Support Vector Machine with the RBF kernel (a popular kernel function used in various kernelized learning). This classifier trains the high-level weights using instances of the target domain, as illustrated in Step 2 of Figure 1. Feeding SMM instances (of the target domain) are fed into low- and mid-level features of the transfer learning framework resulting in output features which serve as input to train the SVM classifier (knowing that all SMM instances are labeled).

## 4. Experimental Setup and Architecture

We conduct our experimental studies on SMM recognition using a dataset of stereotypical motor movements of atypical subjects as well as a dataset of basic activities of typical subjects. Both of these datasets are described in Section 4.1. Processing of both datasets is shown in Section 4.2 based on the preprocessing discussed in the previous section. Next, the CNN architecture structure is described (Section 4.3). Then, the chosen performance indices are defined (Section 4.4).

### 4.1. Dataset Characteristics

“*SMM dataset*” refers to the dataset of stereotypical motor movements for atypical subjects, whereas “*HAR dataset*” (Human Activity Recognition dataset) refers to the dataset of basic activities for typical subjects.

*SMM Dataset.* We perform SMM recognition on a dataset that was collected and released by the authors, Goodwin et al. [7]. In this dataset, accelerometer signals are collected from 6 subjects with autism in a longitudinal study. The data were collected in laboratory and classroom environments. Subjects wore three 3-axis wireless accelerometer sensors and engaged in SMMs (body rocking, hand flapping, or simultaneous body rocking and hand flapping) and non-SMM behaviors. The sensors were worn on the left wrist and right wrist using wristbands and on the torso using a thin strip of comfortable fabric tied around the chest. To annotate the data, subject activities were recorded with a video camera and analyzed by an expert. The first data collection (here called “Study1”), was recorded by MITes sensors at 60Hz sampling frequency. The second dataset (“Study2”) was collected on the same subjects three years later by Wockets sensors with sampling frequency of 90Hz. So, to equalize the data, Study1 data are resampled to 90Hz as explained in the previous section. Data for each subject in each study were obtained in multiple sessions until researchers observed at least 50 SMMs per individual or until they completed three observation sessions per study [7]. An observation session can last 9 to 39 minutes. Two to three sessions were recorded per participant, except subject 6 who was observed only once in Study2. So, we combine data from all sessions for each participant in each study and perform k-fold cross-validation such that k is the number of sessions a participant was observed within each study, and every fold consists of data from a specific session. The purpose is to analyze intersession variability of different SMMs.

*HAR Dataset.* The dataset employed to train the CNN for the HAR task is the PUC dataset [28]. Data was collected from 4 triaxial ADXL335 accelerometers, respectively, positioned in the waist, left thigh, right ankle, and right arm, during 8 hours of activities. The data recorded corresponds to 5 different activities: “sitting”, “sitting down”, “standing”, “standing up”, and “walking”. With a sampling frequency of 8Hz, a total of 165 633 samples were collected. For the sake of our study, we train our deep learning model based on acceleration signals of the waist accelerometers only, since the other accelerometers (located at the thigh, ankle, and arm) will not be relevant to the SMM recognition task during transfer learning. We consider the waist location to be quite similar to the torso location and to be a good tool to detect SMM activities.

### 4.2. Data Preprocessing

#### 4.2.1. Resampling

*SMM Dataset.* Input data are acceleration signals coming from two different types of accelerometers (sensors): one that measures data at a rate of 60Hz and the other at 90Hz. So the 60Hz signals need to be resampled and interpolated to 90Hz. Next, data of both sensors go through a high pass filter with a cut-off frequency of 0.1Hz in order to get rid of noise (DC component).

*HAR Dataset.* Measurements have been recorded at a rate of 8Hz. So, signals need to be resampled from 8 to 90 in order to generate a CNN suitable for SMM input signals. Resampling is done by applying an antialiasing FIR low-pass filter to the signals and compensating for the delay introduced by the filter.

#### 4.2.2. Extraction of Time-Domain Signals

For each subject *i* in study *j*, raw data taken from record sessions are segmented with one-second windows, and the overlap rate between consecutive data segments is set to 88.9%, which means that 90 time-point samples are obtained using a sliding window that moves with 10 time steps (1/9^th^ of a second). Thus, the extracted raw data consists of a vector of length 90 for each coordinate (x, y, and z) per accelerometer. We end up with a 90 × 1 × 9 × *N* matrix for the SMM dataset (9 = 3  *accelerometers* × 3  *coordinates*) and a 90 × 1 × 3 × *N* matrix for the HAR dataset (3 = 1  *accelerometers* × 3  *coordinates*) where *N* corresponds to the number of samples per dataset (i.e., par subject per study). The number of samples (*N*) per subject and per study is displayed in Table 1.

#### 4.2.3. Extraction of Frequency-Domain Signals

We derive ST for every other 10th sample, which at 90Hz is equal to 1/9th of a second. The basic question is how to choose the best frequency range. According to [29, 30], all measured body movements are contained within frequency components below 20Hz. And, as explained in [8], human activity frequencies are between 0 and 20Hz, and that 98% of the FFT amplitude is contained below 10Hz. Accordingly, we first chose the resultant ST frequencies to be in the range of 0 to 10Hz, which did not produce very satisfying classification results. And, after considering Goodwin's observation that frequencies in the range of 1-3Hz covered almost all SMMs [7], we chose a new frequency range of 0 to 3Hz, which produced higher classification rates. So, for every input signal, we compute the ST to obtain the power of 50 frequencies (*τ* = 50) in the range of 0-3Hz, resulting in a feature vector of length 50. Thus, the extracted data consists of a vector of length 50 for each coordinate (x, y, and z) per accelerometer. The resultant input matrix is 90 × 1 × 9 × *N* matrix for the SMM dataset and a 90 × 1 × 3 × *N* matrix for the HAR dataset. The number of samples (*N*) per subject and study is displayed in Table 1.

### 4.3. CNN Architecture Structure

In this paper, several CNN architectures have been conducted, which will be detailed in the next section. However, in order to explain our methodology, the best time and frequency-domain architectures are explained below.

#### 4.3.1. Frequency-Domain CNN

**Figure 2:**
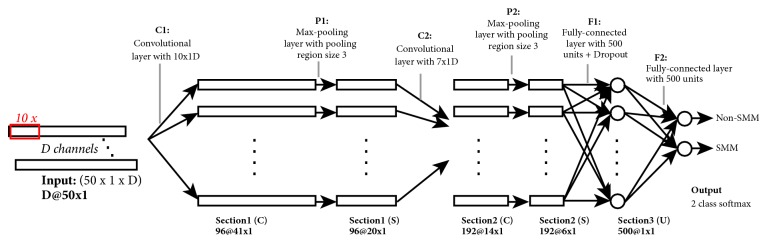
Illustration of the overall frequency-domain CNN architecture. Symbols “*C*”, “*S*”, and “*U*” in the parentheses of the layer tags refer to convolution, subsampling, and unification operations, respectively. Numbers before and after “@” refer to the feature map size. Note that ReLU layers located after* C1*,* C2,* and* F1* are not shown due to the limitation of space.

The overall framework for the frequency-domain CNN is summarized in Figure 2, where two convolutional layers (*C*1, *C*2) and pooling (*P*1, *P*2) layers are stacked on top of one another to form a deep neural network architecture. The input signal, which consists of a data instance, is a 50×1×D matrix, where D corresponds to the number of channels (x, y, and z signals of the used sensors). The first convolutional layer *C*1 filters the *D*-channel 50 × 1 input signal (i.e., 50 × 1 × *D* input matrix), with 96 kernels of size 10 × 1, followed by 3 × 1 subsampling *P*1 (with a stride of 2). The second convolutional layer *C*2 takes the output of the first subsampling layer as input and filters it with 192 kernels of size 7 × 1, followed by 3 × 1 subsampling *P*2. The fully connected layer *F*1 vectorizes the output of the second subsampling layer into a 500-dimensional feature vector. Then, another fully connected layer *F*2 is added followed by a softmax layer.

#### 4.3.2. Time-Domain CNN

Compared to the input vector of length 50 used in the frequency-domain, an input vector of length 90 is employed in the time-domain. Therefore, another CNN architecture is used. Three convolutional layers are needed instead of 2. The first convolutional layer filters the *D*-channel 90 × 1 input signal (i.e., 90 × 1 × *D* input matrix) with 96 kernels of size 9 × 1, followed by 3 × 1 subsampling (with a stride of 2). The second convolutional layer takes the output of the first subsampling layer as input and filters it with 192 kernels of size 7 × 1, followed by 3 × 1 subsampling. The third convolutional layer takes the output of the second subsampling layer as input and filters it with 300 kernels of size 5 × 1, followed by 3 × 1 subsampling. The fully connected layer vectorizes the output of the third subsampling layer into a 500-dimensional feature vector. Then, another fully connected layer *F*2 is added followed by a softmax layer.

### 4.4. Index of Performance

To evaluate the effectiveness of the proposed model, we use the accuracy as our first performance index. And, knowing that the SMM dataset is an unbalanced dataset containing highly unbalanced samples in the test set (with typical movement events being much more frequent than SMM events), we choose the F1-score to be our second performance index.

## 5. Experiments and Results

### 5.1. Selecting Deep Learning Parameters (Experiment 1)

The goal of this experiment is to come up with the optimal value of hyperparameters of the CNN model. The input data used for this study is only a subset of the “SMM dataset”. We choose one of the two studies (Study1) and one of the 6 subjects (Subject1). Accelerometer data of Subject1 in Study1, which are collected from 2 sessions, are used for training and testing in a 2-fold manner; i.e., half of the data is used for training and the other half for testing.

As explained in the methodology, some parameters like the feature map size are tuned using trial-and-error process while some others, such as the filter size of convolutional layers and the number of hidden layers, are determined based on the initial configuration of the input. Accordingly, we investigate the variation of these parameters on the performance of the CNN.

**Figure 3:**
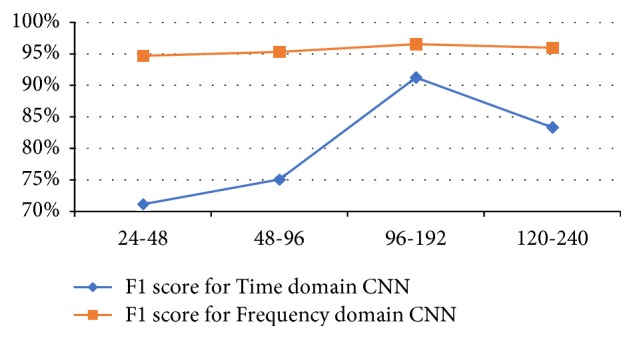
Effect of the feature map size on the performance of SMM classification for the time and frequency-domain CNNs on the testing data. The “24-48” configuration has 24 and 48 feature maps in the 1^st^ and second layers, respectively, and so one.

*Feature Map Size Per Layer.* In this study, we implement the trial-and-error process by considering different time and frequency-domain CNN architectures, in which the size of feature maps is varied between values {24, 48, 96, 120} for the 1^st^ convolutional layer and {48, 96, 120, 240} for the 2^nd^ convolutional layer. The variation of the feature map size for each layer is applied for both the time-domain and frequency-domain architectures and results are displayed in Figure 3. We notice that, as the number of feature maps increases, the SMM recognition rate of the network improves. Indeed increasing the feature map size from 24 and 48 in the 1^st^ and 2^nd^ layer, respectively (“24-48” in Figure 3), to 96 and 192 (“96-192” in Figure 3) increases the F1-score dramatically from 71.14% to 91.23% for the time-domain CNN and increases slightly from 94.71% to 96.54% for the frequency-domain CNN. It is worth mentioning that the size of the input in time-domain CNN is 90 × 1 × 9 compared to 50 × 1 × 9 for frequency-domain CNN. This implies that time-domain CNN needs more feature maps than the frequency-domain CNN in order to capture all low-level and mid-level features present in the input acceleration signals, and 24 feature maps in the 1^st^ convolutional layer are not enough for the time-domain CNN to represent and extract all relevant features.

*Filter Size of the 1st Convolutional Layer*. As explained in the methodology, the first step to determine the filter size of this layer is to find the median of peak lengths of all signals by computing the median of 30 peak lengths collected from 30 random signals (within the dataset) that presented a peak.

**Figure 4:**
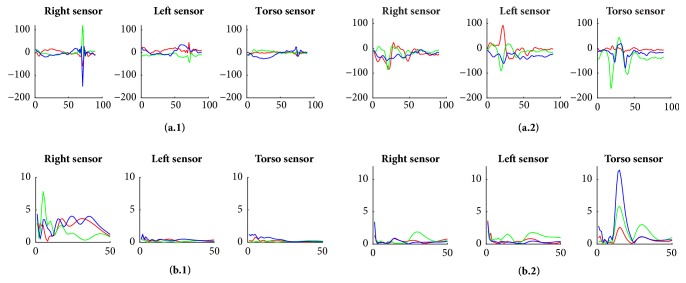
Plots ((a.1)-(a.2)) and ((b.1)-(b.2)) correspond to time and frequency-domain acceleration signal samples, respectively, which are extracted from the “SMM dataset”. The red, green, and blue curves represent x, y, and z signals, respectively. Samples ((a.1)-(a.2)) and ((b.1)-(b.2)) have lengths of 90 (~ 1 second) and 50 (~50 points interval representing frequency in the range of 0-3Hz), respectively.

Before collecting these 30 signals, let us first examine the “SMM dataset” signals.  Figure 4(a) displays 2 samples that correspond to 2 time-domain acceleration signals while Figure 4(b) shows 2 acceleration frequency signal samples; i.e., signals from “SMM dataset” converted into frequency signals via the S-Transform [23]. Each of these samples has x, y, and z signals (represented by red, green, and blue curves, respectively) of the right wrist, left wrist, and torso sensors (represented by the left, middle, and right plots). For each signal, the x-axis stands for the length/duration of the time signal in (a) and of the frequency signal in (b) while the y-axis stands for the acceleration value of that signal. All of these samples are labeled as an SMM activity. In fact, (a.2) is a flap-rock SMM activity due to the presence of high peaks in almost all three axes of right wrist, left wrist, and torso signals. Meanwhile, (a.1) and (b.1) are flap SMM activities with fluctuations in axes of the right wrist signal only, and (b.2) is a rock SMM movement with a variation in torso signals only. The frequency-domain samples (b.1) and (b.2) which have a length of 50 frequency-points show several variations and heights in frequency amplitudes that are 10 to 15 points long (~ a frequency span of 0.2Hz).

Meanwhile, the time-domain samples (a.1) and (a.2), which have a length of 90 time-points, show peaks contained within an interval of 7 to 20 time-points. Thus, observing these time and frequency signal samples gives us a rough idea about the range of the peak duration but cannot provide us with the optimal peak length that will cover most of the peaks within “SMM dataset”.

**Figure 5:**
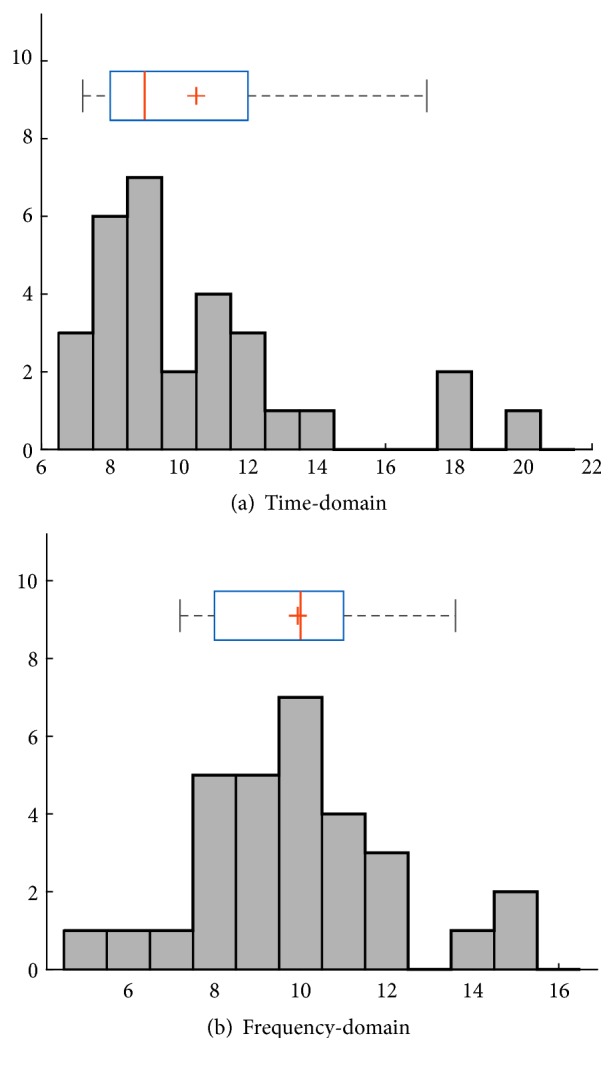
(a) and (b) show histograms and box plots which represent the frequency distribution of 30 peak lengths present within 30 randomly selected time-domain signals and 30 randomly selected frequency-domain signals, respectively.

To that end, we adopt the sampling method by collecting 30 peak lengths from 30 randomly selected signals that present a peak for time and frequency-domains, as illustrated in histograms (a) and (b), respectively, in Figure 5. Histograms (a) and (b) represent the frequency distribution of 30 peak lengths that were present within 30 randomly selected time and frequency-domain signals, respectively. Computing the median of these peak lengths gives 9 in time-domain and 10 in frequency-domain, which represent the optimal filter size of the 1^st^ convolutional for the time and frequency-domain CNNs, respectively.

**Figure 6:**
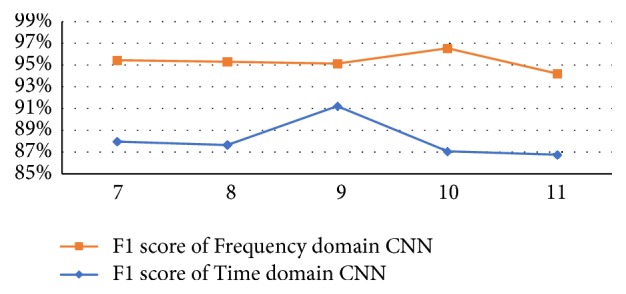
Effect of the size of 1^st^ convolutional layer kernels on the performance of SMM recognition on the testing data.

In order to prove that these calculated values are the optimal filter sizes, we study different time and frequency-domain CNN architectures by varying the value of the filter size. Varying the size of the 1^st^ convolutional layer filter between 7 and 11 across both architectures (time-domain CNN and frequency-domain CNN) confirms the superiority of these computed values, as depicted in Figure 6. In time-domain, an increase in the size of the 1^st^ convolutional layer filter from 7 (~ a time span of 0.078 seconds) to 9 (~ a time span of 0.1 seconds) results in a better classification score since the F1-score goes from 87.97 to 91.23%, suggesting that a larger filter seems to capture more low-level details from the input signal. On the other hand, applying a smaller filter, 10 (~ a time span of 0.11 seconds) and 11 (~ a time span of 0.12 seconds), diminishes the performance of the network. Therefore, 0.1 is the best time span of the 1^st^ convolutional layer that could retrieve the whole acceleration peaks and the best acceleration changes, and the sample median of peak lengths (*M*_*e*_(**x**) = 9) is the optimal peak length and is the proper size for the 1^st^ convolutional layer filter.

In frequency-domain, rising the size of the 1^st^ convolutional layer kernel from 7 (~ a frequency span of 0.14Hz) to 10 (~ a frequency span of 0.2Hz) gives a slightly higher F1-score. However, enlarging the kernel diminishes the performance of the framework. These observations prove that the sample median (*M*_*e*_(**x**) = 10) is the best kernel size for capturing the whole amplitude peak.

*Depth of the Network.* Because of the high dropout rate, it is mostly important to choose the number of layers large enough. And given the small size of the input signals (90 and 50 points in time and frequency-domains, respectively) and the kernel size of the 1^st^ convolutional layer (9 and 10 in time and frequency-domains, respectively), the maximum number of layers that could be stacked is 5 in the time-domain CNN (3 convolutional and subsampling layers and 2 fully connected layers) and 4 in the frequency-domain CNN (2 convolutional and subsampling layers and 2 fully connected layers).

### 5.2. SMM Detection within Subjects with Randomly Initialized CNNs (Experiment 2)

#### 5.2.1. CNN Training

This experiment consists of training one time-domain CNN and one frequency-domain CNN per subject per study. Training is conducted using half the record sessions of an atypical subject *i* for a study *j* from the “*SMM dataset*”. Then testing is implemented using the other half. In other words, feature learning that is performed is specific to one domain (time and frequency), one subject *i*, and one study *j*. The goal of this experiment is to build deep networks that are capable of recognizing SMMs across multiple sessions within the same atypical subject *i*. Both time and frequency-domain CNN architectures are implemented based on the optimal parameters obtained in experiment 1. The other learning parameters are set according to Table 2. Training is performed for 10 to 40 epochs, depending on when the error rate stabilizes and no longer decreases. The dropout is set on a high value (0.5) since training a CNN on instances of the same atypical subject pushes the network to overfit.

#### 5.2.2. CNN Performance

Results achieved by training a CNN for every subject in every study are summarized in Table 3. Training a time-domain CNN on SMMs for each atypical subject yields a good overall performance of 84.59%. So, even with noise present in SMM time-signals, the time-domain CNN is able to capture relevant features for SMM detection. Meanwhile, training a frequency-domain CNN on SMMs for each atypical subject results in an overall F1-score of 93.45%. Thus, time and frequency-domain CNNs provide a good recognition of SMMs across different record sessions within the same atypical subject. Furthermore, comparing between time-domain CNN and frequency-domain CNN demonstrates the efficiency of frequency in detecting SMMs. Indeed, by converting the time-domain signal into a frequency-domain signal, we decompose it into various frequency components. And, by selecting the proper range from 0 to 3Hz (which corresponds to the range of SMMs), all the high frequencies and noisy data are eliminated. Even though the information content in a temporal point is greater than that in amplitude measurements at one source frequency, the magnitude of each frequency has the necessary information needed to perform classification. Moreover, frequency-domain CNN are computationally less expensive than time-domain CNN (450 as input size instead of 900 in time-domain and 2 convolutional layers instead of 3).

#### 5.2.3. Comparison with Other SMM Detection Techniques

Next we evaluate the performance of our proposals against widely used SMM detection techniques, namely, Support Vector Machines (SVM) and Random Forest with Recurrence Quantification Analysis (RF-RQA), as well as a deep learning approach based on CNNs which was proposed by Rad et al. [21]. Also, other machine learning techniques such as Deep Belief Networks (DBNs) are used for comparison. So the first part of this section is to layout their training characteristics of these techniques. The second part compares results of our models to state-of-the art techniques.

*Training*. For SVM, training is conducted using combined performance of both baseline and Stockwell features in the work of Goodwin et al. [7]. We refer to this work as “SVM-C”. Both SVM-C and RF-RQA [8] apply the same dataset as ours and results are already available. As for the DBN, training needs to be conducted. The DBN is first pretrained in an unsupervised manner. Next, an additional feed-forward layer is used to read out the top-level internal representations of the hierarchical generative model. In the unsupervised phase, learning parameters are set as follows: 450 and 810 input vector in frequency and time domains, respectively, 25 hidden units per layer, 0.001 and 0.0001 as the learning rates of the unsupervised (pretraining) and supervised (training) phase, respectively, 0.7 as the momentum in pretraining, 0.3 as the dropout in the supervised phase, 100 as the minibatch size, and 100 and 250 epochs for the unsupervised (pretraining) and supervised (training) phase, respectively. As indicated in [31], a 1-step contrastive divergence learning is applied.

*Results and Analysis.* Table 3 summarizes accuracy and F1-score results of previous works [7, 8, 21], DBN results, and our CNN (in both time and frequency-domains) results. As seen in Table 3, there is a big gap between accuracy values and F1-scores, which is due to the highly unbalanced sample in the test set. Hence, further evaluations will be based on the F1-score measurements. The following observations are made:

- features learned from standard movements of normal individuals (such as walking, sitting, standing, jumping, and running) taken from everyday life basis as the second source domain, which are then applied to recognize SMMs via knowledge transfer. Therefore, this latter is a process performed across two different but related domains, where the source domain is composed of simple human activities of typical subjects and the target domain of SMMs of atypical subjects.
- Time and frequency-domain CNNs outperform by far traditional hand-crafted feature methods (SVM-C) [7] and RF-RQA [8]), which means that deep networks are able to capture a better feature representation from input signals for both time and frequency-domain.
- Time and frequency-domain CNNs perform better than the time-domain CNN work of Rad et al. [21] by 29.82% and 38.68%, respectively, in terms of the mean F1-score. Furthermore, the comparison between results achieved by [21] and our time-domain CNN illustrates the importance of parameterizing neural network attributes properly.
- The time-domain DBN does not yield satisfactory results and does not even converge to a local minima, which is why the corresponding results were not displayed in Table 3. The reason is that neurons of each hidden layer in the DBN learns only variations/peaks of the SMM signal acceleration and does not learn the place of these peaks in the signal since it does not have the concept of ＇local signal patch＇ inside weights as in CNNs. So, as opposed to CNNs which use the ＇weight sharing＇ concept, DBNs are unable to perform well on data that is not aligned by means of size and translation. Since signal acceleration in time-domain presents random peaks that are not translation-invariant across time, the DBN cannot be applied on time-series signals.
- However, SMM signal acceleration converting to frequency-domain amplitudes is translation-invariant across frequency. Therefore, the DBN performs well on frequency-domain input signals. As depicted in Table 3, the frequency-domain DBN achieves an overall F1-score of 82.03%, which is a good score for model as simple and fast as the DBN. Indeed, it detects SMM features (within each atypical subject) which are more representative than those extracted from more complicated approaches such as Recurrence Quantification Analysis [8] and those extracted from computationally heavy approaches such as applying SVM to both baseline and Stockwell features [7]. Meanwhile, the DBN performs less than time and frequency-domain CNNs by 2.57% and 11.42%, respectively, in terms of the mean F1-score.

#### 5.2.4. Feature Representation

**Figure 7:**
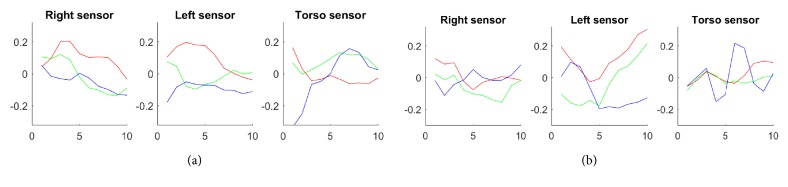
Plots (a) and (b) representing weights of two filters contained in the 1^st^ convolutional layer of the frequency-domain CNN model. The red, green, and blue curves represent weights of x, y, and z signals, respectively.

We analyze the type of frequency features learned by the filters of the first convolutional layer within the trained CNN by examining the learned weights. Let us consider some sample filters of the 1^st^ convolutional layer. Plots (a) and (b) of Figure 7 represent the best 2 out of the 96 1^st^ convolutional layer filters which are chosen based on the highest activation (weights across x-, y-, and z-axis combined). Each of the plots (a) and (b) has nine subplots: the three subplots on the top, middle, and bottom represent the weights of the x-, y-, and z-axis of the right, left, and torso sensor, respectively. We can clearly see sharp fluctuations (edges) in plots, conveying that, during the training phase, filters have learned such variations by detecting peaks and subtle changes in the input acceleration signals. Moreover, we notice that weights in the z-axis of the torso sensor show great variations compared to other weights. In instance (a), we can see a high peak in the z-axis of the torso sensor while other weights stagnate or change with smaller peaks. Thus, weights of filter (a) must probably detect rock SMM activities. On the other hand, in filter (b), weights in all axes show variations especially weights in the z-axis of the torso and left sensors and in the x- and y-axis of the left sensor, implying that this filter probably detects flap-rock SMMs.

**Figure 8:**
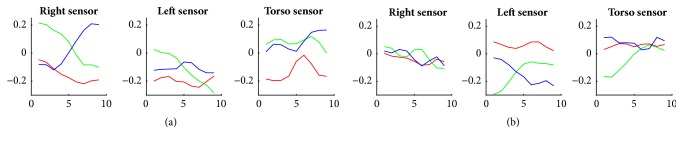
Plots (a) and (b) representing weights of two filters contained in the 1^st^ convolutional layer of the time-domain CNN model. The red, green, and blue curves represent weights of x, y, and z signals, respectively.

We repeat the same process by visualizing some sample filters of the 1^st^ convolutional layer that have the highest activation across all axes x, y, and z (Figure 8). We notice that weights learned in time-domain are different from the ones learned in frequency-domain in different ways: (i) the time-domain weights being less pronounced (having less intensity) than frequency-domain weights, (ii) the time-domain weights having different shapes from the frequency-domain weights with less fluctuations, smoother curves, more downhills (e.g., in the y-axis of the right and left sensor in filter, a, and z-axis of the left sensor in filter, b), and more uphills (e.g., in the z-axis of the right sensor in filter, a, and the y-axis of the left and torso sensor in filter, b). So, this comparison suggests that the CNN learns feature representations specific to each domain (time or frequency). In addition, the smaller intensities, the reduced number of fluctuations, and the smoother curves within the time-domain weights may explain why the time-domain CNN performs less in average (mean F1-score = 87.97%) than the frequency-domain CNN (mean F1-score = 93.23%).

Furthermore, let us note that it is hard to know from Figure 8 whether these filters are responsible for detecting flap, rock, or flap-rock movements. Indeed, we notice small variations in weight intensities in almost all axes within each sensor.

### 5.3. SMM Detection within Subjects with CNN Using Less Input Data (Experiment 3)

We repeat the same process as experiment 2 (CNN in time and frequency-domains) except that input instances are no longer acceleration signals of the three accelerometers (torso, right, and left arm) of a subject *i* (*i* ∈ [1,6]) in study *j* (*j* ∈ [1,2]). Instead, input instances correspond to acceleration signals of the torso accelerometer only while right and left arm sensor signals are omitted. Accordingly, knowing that right and left arm sensors are responsible for detecting “flap movements”, which is a special type of SMMs, and that flap movement instances represent only 5% of the total instances, we choose to omit these flap movement instances as well. Thus, the input matrix will be *D*  ×  1  ×  3  ×  *N* instead of *D*  ×  1  ×  9  ×  *N* where *D* is the length of each input instance and 3 the new channel corresponding to the x, y, and z measurements of the waist sensor. As in experiment 2, we conduct this experiment on every subject *i* in every study *j*. We refer to this experiment as “*Simple CNN*”.

Experimental results are illustrated in Table 4. Comparing the experiment mean classification F1-scores (77.79% and 83.83% in the time and frequency-domain, respectively) to the ones of experiment 2 (84.59% and 93.45% in the time and frequency-domain, respectively) shows that the CNN performs less when acceleration signals of right and left arm sensors are removed. So, these latter hold important information which is relevant to the identification of SMMs, especially flap-rock SMMs that constitute 1/3 of total SMM instances. However, working with an input matrix *τ* × 1 × 3 × *N* instead of *τ* × 1 × 9 × *N* reduces dramatically the complexity of the model with less learning parameters and, thus, less memory, less computational time per epoch, and less epochs to reach the equilibrium (i.e., the maximum classification rate).

### 5.4. SMM Detection across Subjects via Knowledge Transfer

Time and frequency-domain CNNs of experiment 2 adapt to SMMs of new record sessions of one specific atypical subject only and do not adapt to SMMs of another subject since they fail to recognize them, these SMMs being different from one atypical subject to another. Throughout this section, we show that (i) our within-domain transfer learning framework (i.e., within the same domain) can be regarded as a global, fast, and light-weight SMM detection platform that identifies SMMs of a new subject given only few of its labeled SMMs and (ii) our cross-domain transfer learning framework holds the same properties of the former framework with the advantage of being more global and more general. Corresponding results are displayed in Table 4.

#### 5.4.1. Within-Domain Transfer Learning Framework (Experiment 4)

As we know, there is a SMM intervariability across subjects and, for example, testing the CNN of experiment 3 on SMMs of a new atypical subject gives a poor recognition score with an F1-score less than 30%. In other words, features of a CNN trained on SMMs of one subject differ from the ones of a CNN trained on SMMs of another subject. So, we have to train a CNN for each and every atypical subject, requiring computational time and a large SMM dataset per subject, which is quite demanding. In this sense, the purpose of this experiment is to search for differences and similarities in features from one atypical subject to another and explore the relationship between them. First, we assume that high-level features vary from one atypical subject to another and are the ones responsible for these differences; then, we want to find out whether low and mid-level features learned by the CNN (in both time and frequency-domain) are shared across all atypical subjects.

To do so, first, a randomly initialized CNN is trained on subject *i* where *i* ∈ [1,6], for 5 to 10 epochs, using SMM instances of all 6 atypical subjects within study *j* except subject *i*. This process (summarized in Step 1 of Figure 1) results in a CNN with learned weights. Second, low- and mid-level weights learned from this CNN are employed while high-level weights of the last fully connected layer of this CNN are removed and replaced by the SVM classifier. The SVM of the transfer learning framework is trained using a small subset of subject *i*'s training data (i.e., 2000 SMM samples), which results in learned high-level features. Then, the remaining SMMs of subject *i* are implemented for testing the framework. Knowing that the input consists of only a subset of the original training dataset of subject *i* (~ 10000 to 30000 instances), we choose to run the SVM for 5 runs, with 2000 randomly selected samples in each run. In such a way, by aggregating F1-scores of the 5 runs, we provide more realistic results. This procedure is applied on every subject *i* in every study *j*. We refer to this experiment as “*Within-domain transfer learning*”.

The transfer learning framework (composed of the pretrained CNN and SVM) is able to identify SMMs at a mean F1-score of 74.50 % and 91.81 % for time and frequency-domains, respectively, compared to a rough average rate of 30% when directly applying the pretrained CNN for classification. We can infer that low- and mid-level SMM features* share *the same information from one subject to another, making the framework suitable for recognizing SMMs across subjects. In other words, once our CNN is trained on SMM instances of multiple subjects, its features can be used along with the SVM as a global framework to identify SMMs of any new atypical subject.

By comparing these results to experiment 3 results, our framework performs 7.97 % higher than “*Simple CNN”* (the pretrained CNN of experiment 3) in frequency-domain. On the other hand, in time-domain, this framework performs 2.88% less than “*Simple CNN”*. And yet, this ensemble is* simpler* and* faster* with (i) updated high-level weights only, (ii) a faster classifier (SVM compared to “*Simple CNN*” which trains a CNN for multiple epochs until convergence) and (iii) fewer resources, i.e., fewer training instances per subject *i* (2000 instances compared to ~10000 to 30000 training instances used in “*Simple CNN*”).

Besides, in real life, for medical diagnoses of the autism disorder, only few SMM instances are available per subject and computational resources are unavailable, which requires a general light-weight SMM recognition framework that detects SMMs of a new subject based on few of his SMM instances. Thus, the framework discussed in this experiment can be regarded as a global, fast, and light-weight SMM detection framework that suits SMMs of any atypical subject.

#### 5.4.2. Cross-Domain Transfer Learning Framework (Experiment 5)

The aim of this experiment is to search for more global low and mid-level features that adapt to SMMs of any atypical subjects. To this end, we study low- and mid-level features of everyday life basic movements (performed by typical subjects), find similarities between these latter and SMM features, and examine whether they are global enough to be applied for SMM detection across subjects. To explore the relationship between basic movement features and SMM features, we first train two CNNs (one in time-domain and another in frequency-domain) using acceleration signals of basic human activities which are extracted from the “HAR dataset”, as depicted by Step 1 (Figure 1). The time and frequency-domain CNNs have the same architecture as experiment 3. In other words, the same CNN architecture and hyperparameters employed to train on SMM datasets are used to train on the “HAR dataset”. Then, the low- and mid-level features of the trained CNNs are used (kept unchanged) while the high-level features (weights of the last fully connected layer) are discarded and replaced by an SVM classifier (Step 2 of Figure 1), which results in our cross-domain transfer learning framework. Next, we randomly select 2000 instances from subject *i*'s training dataset and feed them to this framework in order to train its SVM and come up with learned high-level features. Again, we choose to run the SVM for 5 runs, with 2000 randomly selected samples from the training dataset in each run. This experiment is conducted for every subject *i* in every study *j*. We refer to this experiment as “*Cross-domain transfer learning*”. The purpose of this study is to show the similitude between SMM low- and mid-level features and features of simple movements and prove the existence of global features that could be applied to the SMM recognition task.

Training this framework produces satisfying results with a mean score of 72.29% and 79.78% in time and frequency-domains, respectively (Table 4). So, fixing low- and mid-level features to features of basic movements and adjusting only the high-level features by an SVM seem to give satisfying classification results, which confirms that our framework has engaged feature detectors for finding stereotypical movements in signals. These results, especially the frequency-domains results, indicate that (i) low- and mid-level features of basic movements along with specific high-level features (learned via SVM using a dataset of an atypical subject *i*) hold a relevant representation, suggesting that both human and stereotypical movements may share low- and mid-level features in common and (ii) this framework can adapt to SMMs of any new atypical subject *i*.

**Comparison with experiment **3**: **this framework provides slightly lower classification rates than “*Simple CNN”* by 5.09 % and 4.05% in time and frequency-domains, respectively. Nonetheless, this framework has the same properties as the within-domain transfer learning framework (experiment 4), thereby being faster and computationally less expensive than the CNN of experiment 3 (which consists of training a randomly initialized CNN) and using fewer resources.

**Comparison with experiment **4**: **this framework yields a lower performance than “*Within-domain transfer learning*” by 2.21% in time-domain and 12.02% in frequency-domain. This implies the superiority in the SMM recognition task of low- and mid-level features learned from SMMs over the ones learned from basic human movements. However, the latter features are more global. For time-series, the small rate difference (2.21%) between this framework and the framework of experiment 4 suggests that the low- and mid-level feature space generated by human activities shares common details with the one generated by movements of specific atypical subjects. This is not the case for frequency-domain series, which can be explained by the difference in the frequency range between human activities and SMMs. Indeed, according to [8], human activity frequencies are between 0 and 20Hz, and 98% of the FFT amplitude is contained below 10Hz. So, training the CNN (of experiment 5) on human activity frequency signals from 0 to 3Hz and not from 0 to 10Hz results in incomplete and imperfect human activity features which, combined with the SVM, do not seem to yield good classification results on the recognition of SMMs. If we were to have a new target domain whose signal frequencies are between 0 and 10Hz, training the CNN on human activities with the frequency range [0, 10] and then combining it with an SVM will yield a better performance on this new target domain. Furthermore, let us note that data used for pretraining in this experiment (taken from the PUC dataset) has a poor frequency resolution with a small sampling frequency 8Hz which had to be resampled to 90Hz, whereas data used for pretraining in “*Within-domain transfer learning*” has a high sampling frequency of 60~90Hz.

One advantage of using this framework over frameworks of experiments 3 and 4 is that datasets of human activities are widely available online while SMM datasets are very rare and harder to obtain and to design. This framework not only resolves the problem of lack of labeled SMM data per subject but also alleviates the issue of lack of labeled data within the entire target domain (SMMs), thereby recognizing SMMs with no datasets of multiple atypical subjects (i.e., historical records of atypical subjects) required. Furthermore, this framework can be applied to more general signal classification tasks which experience a lack of labeled data, implying that the shortage of training data in a target domain no longer limits the size and learning ability of CNN models, especially when it is expensive to obtain fully labeled data. An example of these signal classification tasks is the recognition of movement disorders. To recognize a movement disorder whose acceleration signals have the same frequency range as human activities' signals (0 to 10Hz), applying our framework in* frequency-domain *can be more beneficial than our framework in* time-domain*. Inversely, for movement disorders with a variable frequency range different from human activities' range, implementing our framework in* time-domain *will be more appropriate.

## 6. Conclusion

In this paper, we explore the possibility of using deep learning approaches for the automatic recognition of SMM behaviors within and across subjects. To solve the intrasubject variability of SMMs, we designed time and frequency-domain CNN models whose hyperparameters were chosen based on an analysis of the input space, thereby outperforming state-of-the-art works. To solve the intersubject variability of SMMs, we illustrated how our within-domain and across-domain transfer learning frameworks are scalable, fast, and light-weight solutions which (i) adjust to stereotypical behavior patterns of any new atypical subject requiring only few labeled SMMs and (ii) solve the medical issue of the lack of labeled SMMs per subject. Moreover, we showed that, as opposed to the within-domain transfer learning framework, the cross-domain transfer learning framework does not need SMMs for training since training is implemented using a source domain dataset different from the target domain. Thus, the shortage of training data in any medical target domain no longer limits the size and learning ability of CNN models. As a perspective, the time- and frequency-domain cross-domain transfer learning frameworks can serve as a baseline to the recognition of movement disorders with any frequency and with a frequency range [0, 10Hz], respectively.

## Figures and Tables

**Table 1:** Number of samples (*N*) per subject per study after data extraction in time and frequency domains.

	**Study1**						**Study2**					
Subject	S1	S2	S3	S4	S5	S6	S1	S2	S3	S4	S5	S6

Frequency-domain	27134	17314	34814	20994	24133	30111	30652	27594	41004	47239	29802	13642
Time-domain	27117	17296	34796	20976	24115	30093	30625	27576	40986	47212	29784	13633

**Table 2:** Experimental setup for the CNN model.

**Parameter**	**Value**	**Parameter**	**Value**
Size of input vector	50 (frequency) - 90 (time)	Learning rate	0.01

Number of input channels	9	Weight decay	0.0005

Number of feature maps	24~500	Momentum	0.9

Filter size	7x1 ~ 11x1	Dropout	0.5

Pooling size	3x1	Size of mini-batches	150

Activation function	ReLU	Number of epochs	10~40

**Table 3:** Results of our models and previous works [7, 8, 21] on 6 subjects of Study1 and Study2 datasets. For each study, two to three video sessions were performed per subject except for Subject 6 (in Study2) who had only one session recorded; therefore, experiments could not be performed, which is indicated by “_”. Labels “CNN-Rad” and “RF-RQA” refer to the CNN performed by Rad et al. and the Random Forest with Recurrence Quantification Analysis. Abbreviations “Acc.” and “F1-sc.” refer to “accuracy” and “F1-score”, respectively.

	**Experiments**	**S1**		**S2**		**S3**		**S4**		**S5**		**S6**		**Mean**	
		**Acc.**	**F1-sc.**	**Acc.**	**F1-sc.**	**Acc.**	**F1-sc.**	**Acc.**	**F1-sc.**	**Acc.**	**F1-sc.**	**Acc.**	**F1-sc.**	**Acc.**	**F1-sc.**
**Study1**	SVM-C [7]	85.90	73.00	85.30	36.00	94.00	50.00	66.50	73.00	75.10	44.00	87.30	46.00	82.35	53.67
	CNN-Rad [21]	_	71.00	_	73.00	_	70.00	_	92.00	_	68.00	_	94.00	_	78.00
	RF-RQA [8]	83.00	_	89.00	_	93.00	_	91.00	_	80.00	_	88.00	_	87.33	_
	Time-domain CNN	96.55	91.23	88.51	76.76	97.19	84.95	93.34	93.38	92.51	86.41	94.20	95.11	93.71	87.97
	Frequency-domain CNN	**98.80**	**96.54**	**88.93**	**78.41**	**98.89**	**93.62**	**96.56**	**96.46**	**97.77**	**95.74**	**98.33**	**98.58**	**96.55**	**93.23**
	Frequency-domain DBN	91.50	82.41	87.10	78.06	93.73	71.50	93.55	93.63	89.31	81.69	93.72	94.65	91.49	83.66

**Study2**	SVM-C [7]	71.00	43.00	79.00	26.00	99.00	3.00	90.00	86.00	73.00	72.00	_	_	82.40	46.00
	CNN-Rad [21]	_	68.00	_	22.00	_	2.00	_	77.00	_	75.00	_	_	_	48.80
	RF-RQA [8]	80.00	_	69.00	_	99.00	_	95.00	_	85.00	_	_	_	85.60	_
	Time-domain CNN	96.88	95.97	89.53	75.67	99.10	60.17	96.88	91.68	91.69	82.55	_	_	94.81	81.21
	Frequency-domain CNN	**96.95**	**96.07**	**98.28**	**95.27**	**99.79**	**85.03**	**99.31**	**98.03**	**97.11**	**93.88**	_	_	**98.29**	**93.66**
	Frequency-domain DBN	88.60	85.57	88.84	74.09	99.11	62.70	96.91	91.65	94.04	87.92	_	_	93.50	80.39

**Table 4:** Results of experiments 2, 3, and 4 in time and frequency domains on 6 subjects of Study1 and Study2 datasets.

		Study1						Study2						
	**Experiments**	**S1**	**S2**	**S3**	**S4**	**S5**	**S6**	**S1**	**S2**	**S3**	**S4**	**S5**	**S6**	**Mean**

Time domain	Cross-domain transfer learning	71.66	74.40	66.80	90.69	61.87	92.19	81.35	58.13	35.66	88.44	73.98	_	72.29
	Within-domain transfer learning	**75.37**	**76.44**	56.53	**91.74**	63.37	92.76	84.86	62.97	41.60	**93.32**	80.55	_	74.50
	Simple CNN	74.88	74.52	**71.90**	91.56	**64.46**	**93.95**	**87.48**	**67.62**	**50.21**	92.61	**81.96**	_	**77.38**

Frequency domain	Cross-domain transfer learning	74.50	91.56	43.77	93.11	76.03	94.20	85.16	74.67	66.98	93.66	83.99	_	79.78
	Within-domain transfer learning	90.54	**97.22**	**83.86**	**95.24**	**86.19**	**98.45**	**92.71**	**90.49**	84.99	**97.99**	**92.22**	_	**91.81**
	Simple CNN	**94.40**	80.85	60.17	93.32	79.66	94.73	88.59	83.78	**66.67**	94.77	85.29	_	83.84

## Data Availability

The data that support the findings of this study are openly available in the repository ‘traffic_flow_code' at the URL: https://github.com/lsadouk/code_SMMs.

## Supplementary Materials

Supplementary Materials Values used to plot histograms of Figure 5 are given and detailed in the supplementary materials.
