# Supplementary material for: A Novel Deep Learning Approach for Recognizing Stereotypical Motor Movements within and across Subjects on the Autism Spectrum Disorder
Source: Comput Intell Neurosci. 2018 Jul 10;2018:7186762. doi: 10.1155/2018/7186762 (PMC6077579; doi:10.1155/2018/7186762)
Supplement: Supplementary Materials — Values used to plot histograms of Figure 5 are given and detailed in the supplementary materials. [file 7186762.f1.pdf]

Table 1: Length values of the 30 peaks selected from 30 randomly selected signals of the "SMM dataset".

(a) Time-domain

Starting time point	Ending time point	Length
60	69	9
69	77	8
24	33	9
22	36	14
19	30	11
64	72	8
62	70	8
7	18	11
17	29	12
65	85	20
4	13	9
34	47	13
44	56	12
76	85	9
16	28	12
54	72	18
15	23	8
39	47	8
44	51	7
17	35	18
41	52	11
48	55	7
19	30	11
48	57	9
39	47	8
14	23	9
39	46	7
62	72	10
42	52	10
7	16	9
	**Median**	**9**

(b) Frequency-domain

Starting time point	Ending time point	Length
10	20	10
8	18	10
11	20	9
8	17	9
11	26	15
12	24	12
24	39	15
11	22	11
2	12	10
9	19	10
4	12	8
7	16	9
9	17	8
9	18	9
11	21	10
5	13	8
7	18	11
7	12	5
2	8	6
5	13	8
6	17	11
7	19	12
3	11	8
7	21	14
1	8	7
5	15	10
9	21	12
10	19	9
9	19	10
8	19	11
	**Median**	**10**

Table (a) and (b) represent peak lengths taken from 30 randomly selected time and frequency domain signals. From left to right, the three columns of each table stand for the peak starting time point, peak ending time point and peak length of those 30 signals. These peak lengths are summarized in histograms (a) and (b) within Figure 5.
